# Evaluation of the quality of public health services: A study in the Brazilian context

**DOI:** 10.3934/publichealth.2026003

**Published:** 2026-01-06

**Authors:** Renata Pase Ravanello, Kelmara Mendes Vieira, Breno Augusto Diniz Pereira

**Affiliations:** Graduate Program in Public Organizations Management, Federal University of Santa Maria, Santa Maria, Rio Grande do Sul, Brazil

**Keywords:** health quality management, public health services, health service evaluation, health evaluation, PUBLICSERV

## Abstract

This study investigated Brazilians' perceptions of public health service quality using a multidimensional approach with a national sample. A survey involving 1229 respondents from all regions of Brazil was analyzed through descriptive statistics and confirmatory factor analysis to validate the measurement model. Service quality was assessed using the PUBLICSERV scale, comprising seven dimensions: tangible aspects, reliability, relationship, public value, transparency, equality and legality, and satisfaction. The model showed good fit and validity. Findings revealed that most Brazilians perceive service quality as average across the dimensions. Public value received the highest ratings, highlighting recognition of the services' social importance. Tangible aspects and reliability were lowest, pointing to shortcomings in infrastructure and consistency of care. Transparency was evaluated as moderate, while equality and legality reflected perceived fairness. The relationship dimension was rated positively, suggesting empathy and commitment from health professionals. Overall, satisfaction was reported, though tempered by structural and consistency issues. The results emphasize the value of multidimensional assessments in understanding user experiences, suggesting that improvements in infrastructure and consistency are important to strengthen public health delivery, while maintaining the positive aspects recognized by users, such as social relevance, fairness, and professional commitment.

## 1. Introduction

Organizations increasingly recognize the need to enhance service quality management to address current challenges. In the healthcare sector, assessing quality is of utmost importance [1],[2]. Modern medicine has gradually acknowledged the significance of patients' perspectives in understanding the connections between patient satisfaction and quality of life [3].

Health services encompass all activities aimed at promoting, maintaining, monitoring, or restoring health. These services, provided by healthcare professionals in public or private institutions, include prevention, diagnosis, treatment, rehabilitation, and palliative care [4]. Public health services refer to actions and initiatives promoted, regulated, and funded by the state. In Brazil, the public health sector is designed to ensure universal, equitable, and comprehensive access to health promotion, protection, recovery, and rehabilitation, grounded in the principles of social justice, equity, and solidarity.

Public health services are offered free of charge by states and municipalities, thereby fulfilling the population's social and universal rights. The public health sector is broad and complex but often lacks adequate funding and effective resource management. Financial investments remain low given their significance and the extensive access required. Given its importance, Brazilian public health policy necessitates continuous monitoring of the services provided.

In Brazil, the government is responsible for reducing disease risk and ensuring universal and equal access to health-promoting, protecting, and restoring services through social and economic policies. The Unified Health System (SUS) is the primary agent for health promotion, protection, and recovery, as well as organizing and operating public health services. Complementary legislation ensures community participation in managing SUS and the intergovernmental transfer of financial resources.

The Brazilian public health service is guided by three principles: universality, equity, and comprehensiveness. These principles aim to ensure the provision of services to all individuals in need and to continually enhance the dignity of care. In this context, the SUS is structured and guided by constitutional rights, supporting a commitment to justice and equality in accessing public health services. It also guarantees social participation, essential for formulating and implementing health policies, thereby fostering democratization and improving the quality of health services while promoting social well-being. This perspective reinforces that the right to health is fundamental for maintaining quality of life. Nonetheless, despite assured rights to health services, various obstacles remain in fully implementing its principles, notably regional inequalities.

The evaluation of health services has become a frequent topic in policy discussions [5]. In this context, user satisfaction emerges as a significant component of the evaluation process [6],[7]. In public health services, users are central to the evaluation process, particularly when public management prioritizes quality [8]. In fact, some studies have indeed reported user satisfaction as a quality indicator, and user surveys have become one of the main methods for participation, defense, and protection of rights [9].

From this perspective, public health services should seek feedback from users to identify weaknesses and areas for improvement [10]. Receiving feedback from the population, whether positive or negative, contributes to strategic planning and organizational learning, especially when integrated into performance evaluations, supporting the enhancement of services and public policies [11].

One approach to obtaining such feedback is assessing the perceptions of those who directly use these services [12]. Hence, this study aimed to identify how Brazilians perceive the quality of the public health services provided. Understanding users' perceptions of service quality is important for assessing the current situation, planning improvements, and determining what is necessary to achieve public health management objectives.

Despite the acknowledged importance of user-based service evaluation, Brazil still lacks an institutionalized model for continuously assessing public healthcare services. Consequently, empirical evidence regarding users' perceptions in this context is limited. Therefore, this study contributes to narrowing this gap by providing initial evidence on how citizens evaluate the quality of public healthcare services, supporting the development of more systematic and user-centered evaluation approaches.

## 2. Methods

A survey was conducted using a two-section questionnaire. The first section comprised 50 questions designed to assess users' perceptions of the quality of various public health services. The PUBLICSERV instrument was employed to evaluate public service quality [13], encompassing seven dimensions: tangible aspects, reliability, relationship, public value, transparency, equality and legality, and satisfaction. The second section comprised seven questions intended to identify the respondents' demographic profiles.

The population of interest consisted of Brazilian citizens aged 18 and over who had used any public health service in the previous 12 months. Given that 102,440,025 Brazilians are aged 18 or older, and adopting a 95% confidence level with a 3% margin of error, the minimum required sample size was 1067 respondents. The survey was conducted both in person and online, with interviewers previously trained by the research team. Participants were invited to partake in the survey in public settings, particularly at primary healthcare centers and urgent care units, employing a convenience sampling approach, as individuals were recruited based on accessibility and availability. By the end of the data collection period, a total of 1229 valid responses were obtained. The sample included individuals from all five Brazilian regions, with slight variations in proportional representation relative to the population distribution, mainly due to accessibility and resource-related factors during data collection. The research was approved by the institution's Research Ethics Committee (CAAE no. 47531121.9.0000.5346), and all participants provided informed consent.

The primary analytical techniques employed were descriptive statistics and confirmatory factor analysis, which were used to validate the constructs and the final model. The model was assessed using fit indices and the significance of factor loadings. Commonly used indices include *χ^2^*/*df* (chi-square/degrees of freedom), comparative fit index (CFI), Tucker–Lewis index (TLI), root mean square error of approximation (RMSEA), and root mean square residual (RMSR).

To assess convergent validity, the magnitude of the factor loadings, the average variance extracted (AVE), and composite reliability were evaluated. The literature indicates that factor loadings should be at least 0.5, or ideally higher. The AVE assesses the proportion of item variance explained by its respective construct. Items with values below 0.5 are strong candidates for removal from the factor model; AVE values equal to or above 0.5 indicate model convergence [14]. Composite reliability, which estimates internal consistency, is considered adequate when values exceed 0.7.

For the PUBLICSERV evaluation method, the items were first coded according to the respondents' answers (“Completely disagree” = 1, “Disagree” = 2, “Neither disagree nor agree” = 3, “Agree” = 4, and “Completely agree” = 5). The mean response for the items in each dimension was then calculated. Once the mean values for each dimension were obtained, the overall assessment of public service quality was determined by averaging the perceptions across the seven dimensions. Based on these values, quality was categorized as very low (1.00–1.99), low (2.00–2.99), high (3.00–3.99), or very high (>3.99).

Independent samples t-tests and ANOVA were conducted to assess differences in perceptions across groups. The homogeneity of variances was evaluated using Levene's test before performing both t-tests and ANOVA. When the ANOVA indicated statistically significant differences, post hoc comparisons were performed to identify which specific groups differed significantly.

## 3. Results

Table 1 presents the respondents' profiles based on the following variables: sex, age, education level, and individual income.

Most users (60.5%) were female, with an average age of 32 (ranging from 18 to 71 years). The marital status and education levels of participants varied, with most having completed high school (35.8%) and working as civil servants (32.3%). Most participants reported incomes between BRL 1212.01 and BRL 2424.00 (31.8%).

When comparing the study sample to the Brazilian population profile [13], the median age is quite similar, with 35 years in the sample, compared to 37 years nationally. The proportion of respondents with higher education (17.5%) closely mirrors the 18.4% observed in the general population. However, significant differences are evident in income: while 68.1% of Brazilians earn up to two minimum wages, 56.2% of study participants fall within this income range. Thus, although there is a reasonable alignment between the sample and the national population, respondents tend to have comparatively higher education and income levels.

To validate each construct, confirmatory factor analysis was conducted to assess the unidimensionality and convergent validity of the seven dimensions. Initially, the proposed models included all variables based on PUBLICSERV [14]. The results indicated inadequacy, as some fit indices did not meet the minimum recommended values, and the chi-square ratios exceeded the maximum acceptable limit of five.

To improve the model, correlations were established between errors in five of the seven constructs, which made theoretical sense; variables with loadings below 0.5 were removed. According to Fornell and Larcker [15], validity is supported when coefficients exceed 0.5.

Within the *tangible aspects* construct, initially comprising nine variables, variables 7 and 8 were removed as their loadings were below 0.5 for the AVE adjustment. Subsequently, correlations were established between “e1” and “e2”, “e3” and “e6”, and “e5” and “e6”. To adjust the *reliability* construct, correlations were made sequentially between “e15” and “e16”, “e10” and “e14”, “e10” and “e11”, “e12” and “e13”, and “e13” and “e14” due to inadequate indices.

Variable 27 was removed in the *relationship* construct, which initially comprised nine variables, and correlations were established between “e21” and “e22”, “e23” and “e26”, “e24” and “e25”, “e23” and “e24”, “e19” and “e21”, “e24” and “e26”, and “e25” and “e26”. The *transparency* construct initially contained nine variables, of which variables 38 and 36 were sequentially excluded. Subsequently, correlations were established between “e34” and “e35”, and between “e34” and “e40”.

In the *equality and legality* construct, all five initial variables were retained, and only the correlation between “e45” and “e46” was added. The *public value and satisfaction* construct initially presented adequate indices, requiring neither correlations nor the exclusion of variables. After the adjustments, the constructs demonstrated adequate indices (Table 2).

**Table 1.** Respondents' profiles.

**Variable**	**Categories**	***N***	**%**
Sex	Male	483	39.3
	Female	744	60.5
	Prefer not to answer	2	0.2
Age (years)	18–25	284	23.2
	26–35	293	23.9
	36–47	306	25.0
	>47	343	28.0
Marital status	Single	567	46.2
	Married or common-law marriage	520	42.4
	Separated/divorced	90	7.3
	Widowed	37	3.0
	Other	13	1.1
Education	Incomplete elementary education	94	7.7
	Complete elementary education	98	8.0
	High school	327	26.7
	Technical education	107	8.7
	Higher education	214	17.5
	Graduate education	316	25.8
	Did not attend school	69	5.6
Income (BRL)	≤1212.00	308	25.2
	1212.01–2424.00	379	31.0
	2424.01–3636.00	144	11.8
	3636.01–4848.00	89	7.3
	4848.01–6060.00	100	8.2
	6060.01–7212.00	38	3.1
	7212.01–8484.00	27	2.2
	8484.01–9696.00	18	1.5
	9696.01–10,908.00	29	2.4
	>10,908.00	46	3.8
	No personal income	46	3.8

**Table 2.** Adjustment indices for the seven constructs.

**Index**	**Tangible aspects**		**Reliability**		**Relationship**		**Public value**		**Transparency**		**Equality and legality**		**Satisfaction**	

	**IM**	**FM**	**IM**	**FM**	**IM**	**FM**	**IM**		**IM**	**FM**	**IM**	**FM**	**IM**	
*χ²* (value)	460.596	23.553	388.036	112.979	413.922	63.363	4.832		155.680	17.428	103.192	11.381	5.990	
*χ²* (probability)	0.000	0.001	0.000	0.000	0.000	0.000	0.028		0.000	0.001	0.000	0.023	0.050	
*χ²*/degrees of freedom	23.030	3.926	14.372	5.135	15.33	4.874	4.832		11.120	5.809	20.638	2.845	2.995	
Comparative fit index	0.903	0.995	0.936	0.984	0.941	0.992	0.995		0.959	0.994	0.965	0.997	0.999	
Tucker–Lewis index	0.864	0.988	0.915	0.974	0.922	0.983	0.986		0.939	0.979	0.931	0.993	0.996	
Root mean square residual	0.066	0.018	0.056	0.025	0.043	0.016	0.008		0.038	0.016	0.033	0.012	0.008	
Root mean square error of approximation	0.134	0.049	0.104	0.058	0.108	0.056	0.056		0.091	0.063	0.126	0.039	0.040	
Composite reliability	...	0.863	...	0.898	...	0.914	0.741		...	0.834	...	0.863	0.915	
Average variance extracted	...	0.517	...	0.501	...	0.574	0.490		...	0.503	...	0.559	0.730	

Note: IM: Initial model; FM: final model.; *χ²:* chi-square.

After adjustments were made, the constructs demonstrated convergent validity, as indicated by CFI and TLI values above 0.95 and RMSR and RMSEA values below 0.08 and 0.06, respectively. The constructs also exhibited unidimensionality, with standardized residual values below 2.58. The adequacy of the fit between the estimated and observed matrices was confirmed, as the Chi-square statistic was no longer significant.

Composite reliability values exceeded 0.7 for all constructs, and their AVE values were above 0.5, indicating satisfactory reliability. Only the public value construct had its reliability reasonably adjusted; since it originally consisted of three variables, adjustments to further improve it were not feasible. The model was subsequently validated by examining the previously established fit indices.

**Figure 1.**
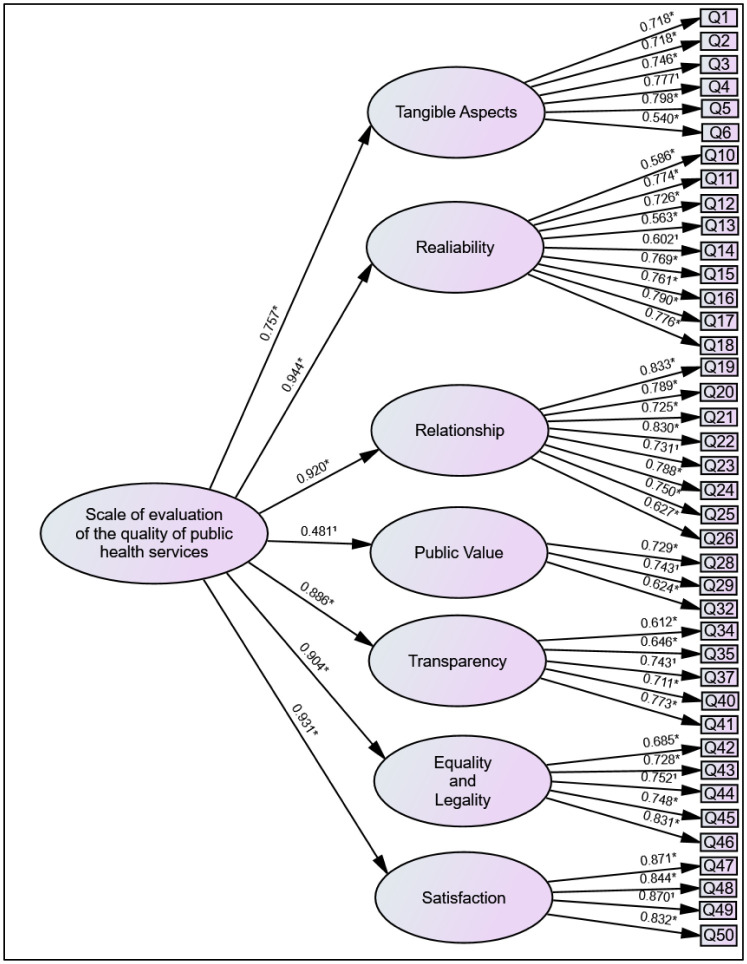
Final model for evaluating the quality of public health services (* *p* < 0.01; ^1^ *z*-value not calculated, where the parameter was set to 1 due to model requirements. For the sake of simplicity, the correlations between the errors were not represented in the figure).

The results indicate that the model is appropriate, as the fit indices demonstrate reasonably adjusted values (Table 3). Values above 0.90 were considered, since indices exceeding 0.90 are desirable [16]. Figure 1 presents the final model.

**Table 3.** Fit indices for the model.

**Index**	**Evaluation of the quality of public health services (second order)**
*χ²* (value)	2892.236
*χ²* (probability)	0.000
*χ²*/degrees of freedom	4.039
Comparative fit index	0.933
Tucker–Lewis index	0.927
Root mean square residual	0.047
Root mean square error of approximation	0.047

Note: *χ²:* chi-square.

**Figure 2.**
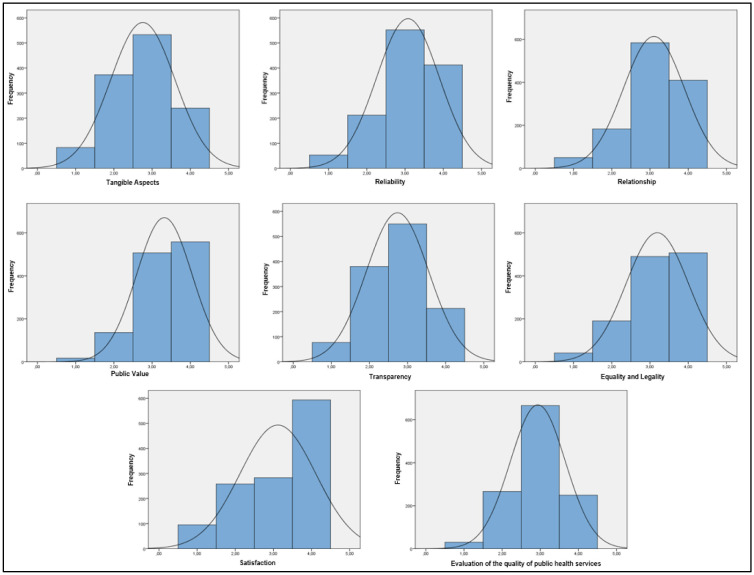
Frequency distribution in each dimension.

The seven dimensions of the PUBLICSERV scale were then analyzed, as outlined by Vieira and Ravanello [14]. Table 4 presents the means and standard deviations, while Figure 2 displays the frequency distribution of the responses.

The mean score is approximately 3.46, indicating that users considered the quality of public health services to be high. For reliability, relationship, equality and legality, and satisfaction, over 70% of users perceived the quality of public health services as high or very high. As for tangible aspects and transparency, users generally provided high ratings, although a considerable proportion evaluated these dimensions as low, in contrast to public value, which was considered very high.

Overall, the general assessment of public health service quality, measured by the PUBLICSERV scale, showed that 75.6% of users considered the quality to be high. Esperidião and Vieira-da-Silva [9] noted that most studies have reported high user perceptions regarding the quality of public health services, regardless of the method employed or the service context.

**Table 4.** Means, standard deviations, and percentages for each dimension.

**Dimension**	**Mean**	**Standard deviation**	**Percentages**			
			**Very low**	**Low**	**High**	**Very high**
Tangible aspects	3.19	0.81	6.8	30.3	43.4	19.5
Reliability	3.53	0.80	4.3	17.2	44.9	33.5
Relationship	3.59	0.78	4.1	14.9	47.6	33.4
Public value	3.70	0.74	1.4	11.2	41.6	45.8
Transparency	3.16	0.79	6.3	31.1	45.1	17.5
Equality and legality	3.59	0.80	3.3	15.5	39.9	41.3
Satisfaction	3.45	1.03	7.7	21.0	23.0	48.3
Evaluation of the quality of public health services	3.46	0.69	2.5	22.0	55.0	20.6

Descriptive statistics for each item comprising the seven dimensions are presented in Supplementary Table S1. These data allow for a detailed analysis of each dimension.

The first dimension (tangible aspects) identifies users' perceptions of the physical characteristics of public health services. Most items received user agreement; however, there was considerable disagreement, particularly concerning whether public health services are well-equipped and adapted to user flow. A significant proportion of users also disagreed regarding the modernity of the facilities. These findings indicate variation among the facilities of public health organizations, which requires adaptation. The subsequent analysis focused on reliability, addressing the extent to which public health services are delivered safely, correctly, and efficiently. Results indicate user perceptions of reliability, though not concerning the adequacy of waiting times.

In the relationship dimension, users reported that staff demonstrated attributes such as safety, responsiveness, and empathy to a moderate degree, suggesting room for improvement to achieve higher quality relationships. Concerning the public service's ability to incorporate public value in meeting users' needs, perceptions of quality were predominant. As for transparency, there was general agreement on the management, quality, and dissemination of information provided by public organizations. Nevertheless, users expressed disagreement about the availability of information.

In the equality and legality dimension, which addresses the treatment received in public health services, most users perceived that their rights as citizens were respected. Lastly, for satisfaction, users were generally satisfied with the public health services and staff and reported that they would recommend the use of the service.

We performed mean difference tests in the final stage of the study to identify differences in the perception of health service quality. Table 5 presents the results of the t-tests, Table 5 also displays the results of the ANOVA.

All tests for differences satisfied the assumption of homogeneity of variances (*p* > 0.05), with the exception of the region variable, for which the hypothesis of homogeneity of variances was rejected (Levene's test, *p* ≤ 0.05). For the variable sex, an independent samples t-test was conducted, revealing no significant differences, on average, in the perception of healthcare service quality. Similarly, no significant differences were found among the Brazilian regions. However, for the remaining demographic characteristics, all results indicated significant differences in group means. Accordingly, Tukey's post hoc test was performed to identify which mean differences were statistically significant.

Regarding age, the youngest group (18–25 years) reported lower perceptions of quality compared to the two older groups (36–47 years and over 47 years). For marital status, a significant difference was observed only between single participants and those classified as married or in a stable union, with single individuals reporting lower perceptions of healthcare quality than those living in a family arrangement.

With respect to education, participants with the highest educational level (postgraduate) reported higher mean scores than those with high school, technical education, and higher education, but similar scores to individuals with elementary education. Lastly, the analysis of income groups indicated that individuals in the two lowest income categories (<BRL 1212.00 and BRL 1212.01–2424.00) perceived higher quality in healthcare services than those in the income group BRL 4848.01–BRL 6060.00 and the three highest income brackets.

**Table 5.** Tests of differences in PUBLICSERV across sex, age, marital status, education, and income.

**Variable**	**Groups**	**PUBLICSERV**		**Levene's test**	**Test of differences**

		**Mean**	**Standard deviation**	**Value (*sig*)**	**Value (*sig*)**
Sex^a^	Male	3.478	0.693	0.307	0.552
	Female	3.453	0.703	(0.580)	(0.581)
Age^b^ (years)	18–25	3.289	0.636		
	26–35	3.425	0.678	1.519	10.229
	36–47	3.547	0.698	(0.208)	(0.000)
	>47	3.563	0.738		
Marital status^b^	Single	3.388	0.703		
	Married or common-law marriage	3.528	0.667	1.48	4.035
	Separated/divorced	3.509	0.776	(0.218)	(0.007)
	Widowed	3.547	0.727		
Education^b^	Incomplete elementary education	3.558	0.746		
	Complete elementary education	3.458	0.669	1.519	4.887
	High school	3.326	0.665	(0.208)	(0.000)
	Technical education	3.418	0.599		
	Higher education	3.377	0.693		
	Graduate education	3.666	0.727		
	Did not attend school	3.368	0.643		
Income^b^ (BRL)	≤1212.00	3.381	0.708		
	1212.01–2424.00	3.352	0.658		
	2424.01–3636.00	3.444	0.685		
	3636.01–4848.00	3.545	0.672	0.724	5.624
	4848.01–6060.00	3.670	0.667	(0.702)	(0.000)
	6060.01–7212.00	3.432	0.713		
	7212.01–8484.00	3.496	0.806		
	8484.01–9696.00	3.925	0.631		
	9696.01–10,908.00	3.937	0.631		
	>10,908.00	3.764	0.727		
	No personal income	3.530	0.729		
Region^c^	North	3.346	0.686		
	Northeast	3.452	0.857	3.766	0.502
	Central-West	3.682	0.747	(0.005)	(0.734)
	Southeast	3.472	0.852		
	South	3.460	0.683		

Note: ^a^ = homoscedastic *t*-test, ^b^ = homoscedastic ANOVA with Tukey post hoc test, ^c^ = heteroscedastic ANOVA.

## 4. Discussion

Public health services are guaranteed to all members of Brazilian society, and the SUS represents one of the most comprehensive and inclusive public health policies. Therefore, assessing the quality of public health services is pivotal given their universality [17]. Furthermore, such evaluation is aligned with the consensus regarding social production and is directly associated with meeting the interests of the user population [18].

In this context, the tangible aspects of public health services were rated moderately by users. These results are primarily negatively influenced by concerns related to physical infrastructure and modernization. Akdere et al. [19] posited that equipment modernization, cleanliness, and presentation are important factors that contribute to users' perceptions of health service quality. Similarly, Marcato et al. [8] reported that users perceive tangible aspects as a major influence on the quality of public health services. Wanjau et al. [20] conducted a study in Kenya and reported that if policymakers understand the interaction between a well-equipped, supportive work environment and the necessary resources for employees to carry out their tasks, both employees and users will experience satisfaction. In Iran, Mosadeghrad [21] showed that the work environment influences satisfaction, emphasizing the importance of creating a welcoming atmosphere.

Reliability was also rated moderately, although most criteria, including legal priorities, meeting needs, and correct service delivery, received positive evaluations. For public health services, user trust is fundamental, and da Silva et al. [17] also inferred that trust enhances the continued utilization of such services by users. Notably, one critical issue within the reliability dimension was waiting time. This perception aligns with studies identifying long waits as a major source of user dissatisfaction, particularly regarding inefficient service flow and appointment scheduling [17]. Similarly, Archibong et al. [22] found that users perceive the quality of health services as low when services are provided in crowded environments, where long queues are a common occurrence.

Regarding the relationship dimension, users perceived empathy, responsiveness, and safety in the health services they received. Akdere et al. [19] observed similar findings, identifying empathy, safety, and responsiveness as key perceptions influencing user assessments of service quality. Nevertheless, a study among Brazilian patients indicated that safety, responsiveness, and empathy should be improved, as users reported low perceptions in these areas [8]. Wanjau et al. [20] emphasized that the quality of public health services is shaped by the capabilities of employees. Erić and Slavković [23] inferred, through a study conducted in health organizations in central Serbia, that satisfaction is enhanced by human resource management policies and practices, which, in turn, impact the quality of public health service outcomes. Mosadeghrad [21] showed that care relies not only on the healthcare professional but also on the cooperation, responsibility, and involvement of the patient, affecting both treatment and their perception of health service quality.

Public value was rated higher by users, primarily due to the recognized need for public resources to sustain these services and their societal importance. From this perspective, the availability of financial resources in public sector hospitals significantly influences the quality of services provided; thus, their management should be enhanced to ensure efficiency [24]. Mosadeghrad [21] highlighted that the most critical factor for public health policymakers and managers is financial resource availability, as it directly affects the quality of health services. Conversely, a scarcity of these resources results in increased stress among employees. Public value concerns the benefits that society derives and what citizens value when processes, services, and outcomes generate value for the public sphere [20]. Research involving public managers and various stakeholders is needed to determine their perceptions and value propositions.

In terms of transparency, users noted partial quality in the management and disclosure of information. Increased and improved public access to state data enhances understanding of public services and facilitates social control [25]. Wanjau et al. [20] inferred that effective communication channels contribute to users' access to treatment, in research conducted at Kenyatta Public Hospital located in Kenya. Thus, the provision of quality health services is influenced by the communication channels used. Thus, it is necessary to adopt more proactive transparency practices, as the mere dissemination of complex and disorganized information does not support effective co-management and oversight of public administration. Such processes should be legitimized by popular participation and based on interaction between public administration and citizens.

In the equality and legality dimension, users considered these principles to be upheld in the provision of public health services. In the public context, the application of constitutional principles is fundamental for citizens to feel that their rights are respected and protected. This guarantee is emphasized in SUS legislation, which ensures access to public healthcare for all and constitutes the primary health policy in Brazil [17]. In fact, evidence from other countries has indicated that the capacity of health service providers to deliver fair and equitable services is among the main determinants of service quality [26].

Regarding satisfaction, users reported being generally satisfied with public health services, thereby corroborating research on satisfaction conducted in Brazil and abroad [27],[28]. Bordin et al. [6] also reported a high level of user satisfaction in their evaluation of public health services, albeit emphasizing the need for caution when interpreting these results, primarily due to the low level of criticism and demands from Brazilian public health users, as well as a degree of conformity with the services received. Similarly, Esperidião and Vieira-da-Silva [9] noted a high perception of satisfaction, noting that users' experiences and health needs may influence their judgment of the services, whether due to reluctance to provide negative feedback or gratitude for having their needs met.

In general, the mean difference tests indicate that younger individuals, particularly those who are single and have an educational level equivalent to high school, technical education, or higher education, as well as those with lower incomes, tend to perceive lower quality in public healthcare services compared to older individuals, those in stable relationships, with higher educational attainment, and higher income levels. According to Mosadeghrad [2], the interaction between healthcare professionals and patients is influenced by sociodemographic factors, mainly concerning patients' knowledge of their rights, which affects their expectations regarding the quality of healthcare services. Furthermore, more educated patients tend to have more realistic expectations of healthcare professionals.

These results suggest that the socioeconomic profile is an important factor in assessing the quality of public healthcare services, as it highlights which groups of citizens report lower average ratings. Identifying these groups and conducting a detailed analysis of the reasons underlying their lower evaluations is a critical step for public healthcare organizations seeking to improve service quality.

When considering all dimensions simultaneously, the perceived quality of public health services is classified as high. Nonetheless, the presence of low-quality reviews should be highlighted, as they indicate key areas for improvement identified by users. While public health services were deemed satisfactory, they were not considered ideal. One promising alternative is the development of a culture of evaluation within public health services.

Effective monitoring requires the establishment of parameters and indicators to assess service quality and effectiveness. Generally, compliance with legal requirements is observed, albeit not with the intention of serving as an evaluative instrument for public management.

Obtaining scientifically valid information on user perceptions of public health services creates new opportunities for service management. Such information also supports the establishment of management priorities. Therefore, by evaluating health services with indicators that enable quality analysis and assessment, it becomes possible to reconsider strategies for action and management in health services [17].

In this context, Gallani et al. [29] highlighted the fundamental importance for public service managers, particularly decision-makers, to disclose users' perceptions. Furthermore, public managers should proactively seek out users' perspectives regarding quality management [30]. Public policymakers and service providers must recognize that enhancing health services is a key aspect for beneficiaries and patients.

Evaluating user perceptions and integrating these findings into organizational management processes is essential for enhancing public healthcare services. This study, for instance, identified waiting time as a significant concern from the users' perspective. Therefore, healthcare organizations should devise targeted strategies, such as optimizing scheduling processes, increasing the availability of service professionals, and expanding access to specialist consultations, to reduce waiting times. Integrating initiatives aimed at addressing this issue into organizational performance goals and operational planning may contribute to more efficient service delivery and improved user satisfaction.

## 5. Final considerations

The provision of public services is increasingly subject to societal demands for higher quality and efficiency [1],[31],[32]. Consequently, conducting assessments of service quality enables managers to understand the effects of specific quality improvement measures, including satisfaction levels and the evolving needs and desires of citizens.

In public health, there is consensus that user satisfaction is multifaceted and difficult to measure [33]. This complexity often leads to surveys producing data that are disconnected from actual experiences or indicating dissatisfaction with the services. Although legal provisions for evaluating public service quality exist, their practical application remains limited. Quality has, nonetheless, long been a concern for healthcare service providers worldwide [34].

Effectively responding to societal demands requires strengthening governance mechanisms. Closer collaboration between the state and society facilitates the governance process and co-production, leading to improvements in the quality of public services delivered. Accordingly, it is essential for service managers to be attentive to the needs of service users.

The principal contribution of this research is its comprehensive examination of public health service quality assessment in Brazil. This study provides significant insights into the dimensions of citizens' perceptions of the quality of public health services. Moreover, research into service quality at the citizen level can establish a foundation for individuals to serve as key participants in the process of understanding, evaluating, and advancing public management practices. The aim was to provide feedback and understand the perceptions of users of public health services so that their needs can be identified and prioritized to inform the development of management improvement strategies. In this regard, Alanazi et al. [34] asserted that public health policymakers must understand and integrate users' perceptions and expectations to ensure positive outcomes in the provision of health services. This is particularly important given the frequent face-to-face interaction with users of these services [35].

This study has sampling and methodological limitations. Regarding the sample, it is important to note the use of convenience sampling and the lack of proportional representation across regions when compared with the population distribution, primarily due to access restrictions and resource limitations during data collection. Additionally, typical constraints associated with survey-based research, such as the potential for socially desirable responses and recall bias, should be acknowledged. These factors may affect the generalizability of the findings and must be considered when interpreting the results.

Consequently, further research should be conducted to evaluate the quality of public health services across different user groups, as well as by type of service and delivery units, to better identify users' primary needs and the necessary improvements to enhance service quality.

Future research should assess the quality of public health services across different user groups, service types, and delivery units to identify users' needs and priority areas more precisely for improvement. Considering cultural, regional, and socioeconomic differences, the proposed model should be applied to diverse samples and contexts, including variations according to the type of medical specialty users demand. Such approaches would facilitate comparative analyses and support the prioritization of actions based on local demands.

Additionally, longitudinal and causal studies are recommended to monitor changes over time and investigate the effects of perceived service quality on outcomes, such as patient satisfaction, loyalty, and the generation of public value within the health system. These studies could provide stronger evidence on the role of service quality as a driver of public value creation, supporting more informed decision-making in health service management.

## Use of AI tools declaration

The authors declare they have not used Artificial Intelligence (AI) tools in the creation of this article.


