# Supplementary material for: Evaluation of the quality of public health services: A study in the Brazilian context
Source: AIMS Public Health. 2026 Jan 6;13(1):32–49. doi: 10.3934/publichealth.2026003 (PMC13084497; doi:10.3934/publichealth.2026003)
Supplement: Supplementary file 1 [file publichealth-13-01-003-s001.pdf]

AIMS Public Health

AIMS Public Health, 13(1): 32–49.
DOI: 10.3934/publichealth.2026003
Received: 21 October 2025
Revised: 07 December 2025
Accepted: 13 December 2025
Published: 06 January 2026

https://www.aimspress.com/journal/aimsph

Research article

Evaluation of the quality of public health services: A study in the Brazilian context

Renata Pase Ravanello, Kelmara Mendes Vieira* and Breno Augusto Diniz Pereira

Graduate Program in Public Organizations Management, Federal University of Santa Maria, Santa Maria, Rio Grande do Sul, Brazil

* **Correspondence:** Email: kelmara.vieira@ufsm.br; Tel: +555532209265.

Supplementary

Table S1. Descriptive statistics of constructs, averages, and percentages.

Constructs	Items	Average	Percentages				
			Totally disagree	Disagree	Indifferent	Agree	Totally Agree
Tangible aspects	Public health body had adequate equipment to provide the service.	3.46	5.0	23.2	4.8	54.5	12.4
	The equipment used by servers of public health body worked properly during the service.	3.68	2.8	16.5	6.1	58.2	16.4
	Physical facilities of public health body were comfortable.	3.11	8.0	30.9	10.1	43.3	7.8
	Physical facilities of public health body were adapted to the services offered.	3.43	4.6	21.5	10.1	53.5	10.2
	Physical facilities of public health body were well equipped.	3.09	6.1	34.0	11.3	42.0	6.6
	Physical facilities of public health body were modern.	2.62	13.4	43.4	15.0	23.7	4.4
	The facilities of public health body were suitable for people with disabilities.	3.21	8.1	25.8	10.7	47.0	8.4
	The physical space in public health body was suitable for the flow of users.	2.92	12.4	33.5	9.5	37.8	6.8
Reliability	Public health body delivered the service within the established period.	3.32	8.8	22.6	8.6	47.5	12.5
	The service of public health body was correctly provided.	3.69	4.1	14.6	5.7	58.8	16.8
	I felt heard by the server that provided the service at public health body.	3.66	5.5	12.6	9.9	54.1	18.0
	The service order at public health body respected the legal priorities.	3.73	3.4	12.9	8.4	57.3	18.0

	The waiting time for assistance at public health body was adequate.	2.77	19.4	33.0	6.2	33.2	8.2
	I felt safe with the information received during the service at public health body.	3.62	4.6	15.4	9.1	54.8	16.2
	I obtained the necessary information regarding the service provided by public health body.	3.66	3.4	15.3	8.0	58.2	15.1
	I trust the service provided by public health body.	3.62	3.7	15.8	10.5	54.0	16.1
	The services offered by public health body met my needs.	3.66	4.6	14.4	7.6	56.3	17.0
Relationship	Servers of public health body were helpful.	3.75	3.7	11.8	9.7	54.8	20.0
	Servers of public health body provided accurate information.	3.62	3.8	15.1	12.0	53.8	15.4
	Servers of public health body were educated.	3.83	2.7	11.0	8.2	56.6	21.5
	Servers of public health body were willing to help me.	3.76	3.0	10.7	12.5	54.2	19.6
	Servers of public health body gave personalized attention to my need.	3.27	6.5	21.1	24.0	35.2	13.2
	Servers of public health body were able to respond to my queries.	3.68	2.7	13.4	11.2	57.4	15.3
	Servers of public health body seemed qualified.	3.69	2.7	14.6	10.3	55.8	16.6
	Servers of public health body tried to help meet my need even when it went beyond their duties.	3.10	7.2	30.5	18.1	33.3	10.9
	I feel that the service I received from the server(s) of public health body is the same as that provided to other people.	3.58	4.1	16.8	10.4	54.0	14.7

Public value	I feel that the public service provided by public health body is important for society.	4.45	0.6	2.7	1.3	41.2	54.1
	It seems fair to me that the government uses public resources to maintain the services provided by public health body.	4.38	1.3	4.2	2.8	38.2	53.5
	During the service, I tried to preserve the public goods made available by public health body.	4.35	0.3	2.5	3.3	49.5	44.4
Transparency	Public health body provides broad access to their information.	3.16	5.5	29.8	15.7	40.9	8.1
	Public health body has efficient ways of communicating with society.	2.93	8.9	35.9	14.8	33.5	6.8
	Public health body provides different ways of requesting the service.	2.93	8.8	35.9	14.4	34.9	6.0
	I received clear information about the service requested from public health body.	3.49	3.6	19.2	13.0	52.8	11.4
	I was notified of the forms of monitoring the progress of the service requested from public health body.	3.32	6.1	21.4	17.8	43.4	11.2
	Information about the services provided by public health body is transparent.	3.03	7.1	32.9	16.3	36.8	7.0
	The forms of assistance used by public health body for the provision of the service are adequate.	3.29	4.7	27.0	10.7	49.4	8.3
Equality and legality	In providing this service, I realized that equal treatment between users is guaranteed by public health body.	3.46	4.7	20.9	11.8	48.8	13.8

	I felt that public health body provided me with a fair service compared to what other users received.	3.53	4.1	16.5	14.0	52.2	13.2
	The service priorities established by public health body were fair.	3.63	3.8	14.3	11.2	56.1	14.6
	Legal aspects involved in the provision of the service by public health body have been complied with.	3.64	3.5	12.0	14.8	56.4	13.3
	I felt that my rights as a citizen were respected in providing the service by public health body.	3.69	4.1	12.7	8.5	58.9	15.7
Satisfaction	I am satisfied with the service received from public health body.	3.36	7.4	24.1	7.9	46.0	14.5
	I am satisfied with the service provided by servers of public health body.	3.49	5.9	20.3	8.1	49.5	16.2
	The service provided by public health body met my expectations.	3.35	7.6	23.2	9.3	45.9	14.1
	I would recommend the services provided by public health body.	3.60	5.8	13.5	15.6	44.7	20.4

AIMS Press

© 2026 the Author(s), licensee AIMS Press. This is an open access article distributed under the terms of the Creative Commons Attribution License (https://creativecommons.org/licenses/by/4.0)
